# Immunosensors—The Future of Pathogen Real-Time Detection

**DOI:** 10.3390/s22249757

**Published:** 2022-12-13

**Authors:** Edyta Janik-Karpinska, Michal Ceremuga, Marcin Niemcewicz, Marcin Podogrocki, Maksymilian Stela, Natalia Cichon, Michal Bijak

**Affiliations:** 1 Biohazard Prevention Centre, Faculty of Biology and Environmental Protection, University of Lodz, Pomorska 141/143, 90-236 Lodz, Poland; 2 Military Institute of Armored and Automotive Technology, Okuniewska 1, 05-070 Sulejowek, Poland

**Keywords:** immunosensors, biosensors, detection, pathogens, biological agents

## Abstract

Pathogens and their toxins can cause various diseases of different severity. Some of them may be fatal, and therefore early diagnosis and suitable treatment is essential. There are numerous available methods used for their rapid screening. Conventional laboratory-based techniques such as culturing, enzyme-linked immunosorbent assay (ELISA) and polymerase chain reaction (PCR) are dominant. However, culturing still remains the “gold standard” for their identification. These methods have many advantages, including high sensitivity and selectivity, but also numerous limitations, such as long experiment-time, costly instrumentation, and the need for well-qualified personnel to operate the equipment. All these existing limitations are the reasons for the continuous search for a new solutions in the field of bacteria identification. For years, research has been focusing on the use of immunosensors in various types of toxin- and pathogen-detection. Compared to the conventional methods, immunosensors do not require well-trained personnel. What is more, immunosensors are quick, highly selective and sensitive, and possess the potential to significantly improve the pathogen and toxin diagnostic-processes. There is a very important potential use for them in various transport systems, where the risk of contamination by bioagents is very high. In this paper, the advances in the field of immunosensor usage in pathogenic microorganism- and toxin-detection, are described.

## 1. Introduction

Pathogens and their toxins possess the ability to adversely affect humans and animals with a range of relatively mild reactions up to severe course of disease, and in some cases can cause death [1,2]. Pathogens include organisms such as bacteria, viruses, fungi and parasites [3,4], and they can be found in numerous environments including water, soil and air [5,6,7]. Pathogens can be transmitted from their natural reservoir to a susceptible host in different ways. The mode of transmission can be characterized on the basis of pathogens spreading in direct and indirect ways. In direct transmission, pathogens are transferred by direct contact or by droplet dispersal. Indirect transmission occurs when pathogens can be transmitted by suspended air particles, inanimate objects (water, food, blood, bedding, surgical equipment, toys, environment), or animate intermediaries (mosquitoes, fleas, ticks) [8,9]. In addition, pathogens possess the ability to rapidly evolve as well as to adapt and grow under different conditions such as low or high temperatures, basic or acidic pH, a wide range of salinities and various pressures [10]. Biological toxins consist of harmful substance produced by various organisms such as: bacteria, fungi, insects, vertebrate and invertebrate animals, and plants, mainly for defensive purposes [2,11]. These molecules can also be present in various environments and induce detrimental effects in other organisms, which can contract them by injection, inhalation or absorption [12]. 

Rapid detection of pathogens and toxins is of the greatest importance primarily for health and safety reasons and reducing the risk of pandemic contamination. The food industry, water and environmental quality control and clinical diagnostics are the main areas where prompt biological-agent detection is crucial [2,13]. The existing methods used to detect pathogens and toxins rely on conventional techniques such as plate culturing, enzyme-linked immunosorbent assay (ELISA) and polymerase chain reaction (PCR) [14,15]. Plate culturing is one of the oldest method of pathogen identification; however, it still remains the “gold standard” for bacteria detection, due to its high sensitivity and selectivity. In spite of this, the culturing technique requires selective plating, several days for enrichment, identification, and confirmation and numerous microbiological procedures that are time-consuming and monotonous [16,17]. Immunological assay ELISA is also used to detect pathogens, and is one of the most popular immunoassay methods. This technique can provide results slightly faster compared to plate culturing, but the high number of false positives and the experimental complexity limit its use [18,19,20]. The molecular-based detection method—PCR, for example—is a very popular technique for pathogens’ detection [21]. Specific pathogens based on their nucleic acid sequence are targeted when PCR is used for detection. PCR can detect a single copy of the target DNA sequence, and thus can be used to detect, for example, a single bacterium in a food sample [22,23]. This method can be used for pathogen detection with high specificity and sensitivity, but requires costly instrumentations, several steps of procedure and well-qualified personnel to operate the whole experiment and to interpret the obtained results [24]. In spite of disadvantages such as the complexity of use or the time required for the analysis, it is still used successfully as an effective detection tool. The above techniques are often combined to obtain more reliable results [13,25,26].

**Figure 1:**
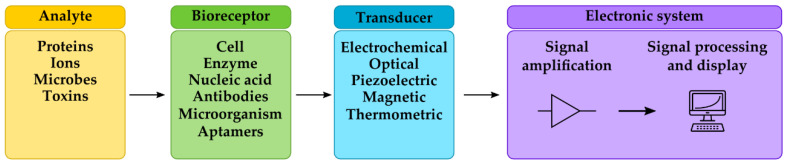
Basic principle and biosensors components.

Despite the effectiveness of conventional methods, there is a need for new technology that is simple, rapid, specific, sensitive and reliable. Moreover, it should be appropriate for in situ real-time monitoring at low cost. In recent years, there has been increased research activity in the field of biosensors development for the detection of pathogenic microorganisms and toxins [27,28]. A biosensor is an analytical device, which integrates a biologically derived molecular-recognition molecule into a suitable physicochemical-transducing mechanism and converts a biological response into an electrical signal [29]. Biosensors consist of two principal elements: a bioreceptor or biorecognition component that recognizes the target analyte, and a transducer that converts the recognition event into a measurable electrical signal (Figure 1). The bioreceptor can be the tissue, cell, enzyme, antibody, nucleic acid, microorganism, organelle and others. Common transducing elements are: electrochemical, optical, piezoelectric, thermometric, magnetic, micromechanical, or combinations of one or more of those techniques [30]. 

Compared to conventional techniques, biosensors do not require highly qualified personnel. Furthermore, if a biosensor is highly selective and sensitive, it can provide results faster than standard methods, making it ideal for practical and field applications [31,32]. This paper aims to give an overview of pathogen and biological-toxins detection using immunosensors. It describes different electrochemical, optical, and piezoelectric platforms for the detection of different pathogens and biological toxins.

## 2. Methods

A literature search was carried out using PubMed and Medline databases. A total number of 137 articles were analyzed, including 80 original research papers and 57 reviews (meta-analyses, systematic reviews, literature reviews). In this paper, we have included articles mainly from the last 10 years. All scrutinized articles focused on biosensors proposed for the detection of pathogens and biological toxins, with particular emphasis on their clinical capabilities and use in point-of-care diagnostics. Moreover, all articles were published in the English language. Search terms included “biosensors”, “immunosensors”, “electrochemical biosensors”, “piezoelectric immunosensors”, “optical immunosensors”, “conductometric platform”, “impedimetric platform”, “potentiometric platform”, “amperometric platform”, “biosensors bacterial diagnostic”, “biosensors viral diagnostic”, “biosensor point-of-care diagnostic”. Therefore, we excluded papers not published in English. Independently, the three authors searched the databases for articles on types and descriptions of biosensors, and three for the clinical application of biosensors.

## 3. Immunosensors

Immunosensors are a type of affinity solid-state based biosensors, in which the target analyte, antigen (Ag), is detected by the formation of a stable complex between Ag and the antibody (Ab) as a capturing agent. This immunological reaction results in the generation of a measurable signal given by the transducer [33]. The action of immunosensors may be similar to immunoassays, but there is a subtle difference between them. The immunoassay test is a solid phase system in which the Ag-Ab complex takes place, but the detection is carried out elsewhere. In immunosensors, interaction between Ab and Ag, and the recognition process of the Ag occur within the same platform [34,35]. Based on their transduction mode, immunosensors can be classified into three main types, including electrochemical (amperometric, potentiometric, impedimetric, and conductometric), and optical and piezoelectric devices [36]. Depending on the transducer type and the signal-processing modes, immunosensors are divided into label-free and labeled sensors [37]. Label-free immunosensors measure the physical or chemical changes resulting from the Ag-Ab immune-complex formation without labeling [38]. Label-free detection reduces the preparation time, sample complexity and analysis cost, and enables detection of target-probe binding in real-time, which is generally not possible with label-based systems [39]. However, a problem with the use of label-free immunosensors can be the non-specific adsorption on their response. In general, in the absence of Ag-Ab interaction, no signal should be observed; nevertheless, a slight signal can always be obtained because of the non-specific Ag or different proteins binding to the substrate’s surface [40]. This phenomenon occurs due to the presence of other proteins in the sample that can adsorb to the Abs or support surface, leading to an increase in the background signal. The consequence of the non-specific adsorption is a decrease in sensitivity. Therefore, it is necessary to use a suitable blocking agent. A number of compounds are used as blocking agents, such as: casein, bovine serum albumin (BSA), and other milk proteins, surfactants (polyethylene glycol, Tween 20), and thionic compounds for gold surfaces [33,41]. Labeled immunosensors use signal-generating labels, such as enzymes (catalase, glucose oxidase), fluorescent dyes, and metal ions, and also nanomaterials, such as gold nanoparticles (AuNPs), carbon dots (C-dots) and quantum dots (QDs) [37,42]. In this type of immunosensor labels can be attached to the Ab or Ag, resulting in electron-transfer and assuming that happens, the number of labels detected during measurement correlates with the number of target analytes [33]. Compared to label-free, labeled immunosensors possess a lower effect of non-specific signal adsorption and higher versatility and sensitivity, due to the analytical characteristics of the applied label. Disadvantages include the inability for real-time monitoring of the Ag-Ab reaction and high operation and development costs [37,40]. Labeled immunosensors can be further divided into two other types of assays: the competitive and the sandwich type, according to the analytes’ molecular size. Competitive-type assays are applied for the small-molecule compounds (e.g., pesticides) with a small molecular weight and only one epitope. The analytes in the samples are measured based on their ability to compete with the labeled Ag in the immunosensors. The signal obtained from the labeled analyte is inversely proportional to the sample amount of the analyte. Thus, the responses decrease as the concentration of the analyte increases [37,43]. Sandwich-type assays are preferred for macromolecular compounds with high molecular weight (e.g., proteins) and more than one epitopes. The detected signal-responses are directly proportional to the number of analytes in the analyzed samples [33,37].

### 3.1. Electrochemical Immunosensors 

**Figure 2:**
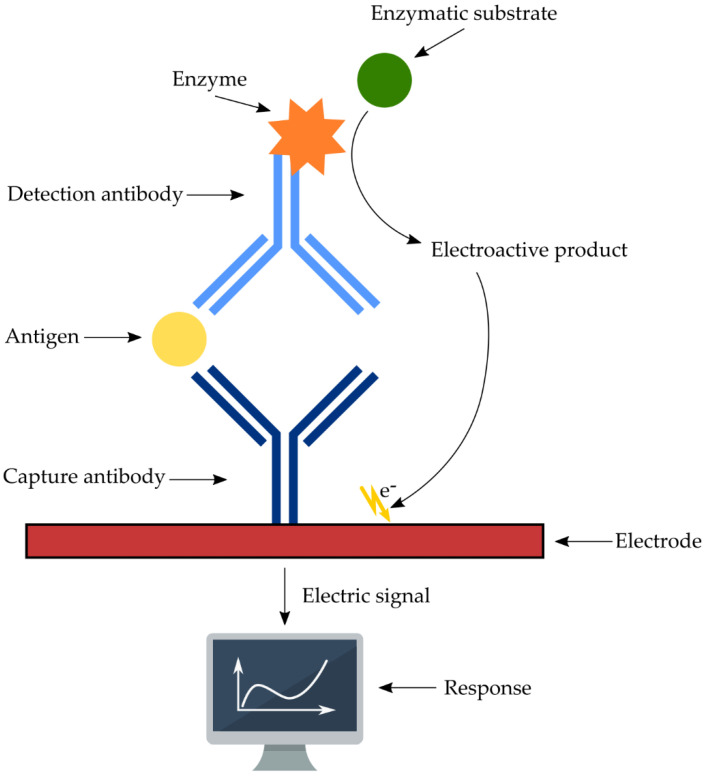
Schematic diagram of electrochemical immunosensor.

Electrochemical transducers are the most commonly used methods in biosensors, and can be broadly classified into label-free and labeled sensor. The principle of this method is based on the selective identification of the Ag (analyte) by the capture Ab immobilized on the electrode surface. The label-free electrochemical immunoassay can determine the concentration of the analyte by direct measurement of the Ag-Ab’s specific recognition of the change in the electrochemical signal which is generated after binding. The sandwich-type electrochemical immunosensor additionally uses the detection Ab, which is often labeled as enzymes or fluorescent labels [44]. The signal is usually the result of a catalytic reaction of the enzyme molecule labeled as a signal tracer with the detection Ab. The electroactive product containing electric charges can be detected by the electrode [45]. The electrode can derive the signal which is generated on the electrode surface and convert it into an electrical signal, including voltage, current, and resistance, which can be measured and analyzed to obtain a qualitative or quantitative analysis of the analyte, e.g., toxin, pathogen, or disease biomarker (Figure 2) [43,46]. In general, electrochemical immunosensors can detect different analytes by measuring the change in potential, current, conductance, or impedance, caused by the immunoreaction. This type of immunosensor can be also classified as amperometric, potentiometric, impedimetric, and conductometric, depending on the type of signal [47].

#### 3.1.1. Amperometric Platform

This method is based on the measurement of a current flow that relates to the concentration of a measured analyte such as a pathogen. The amperometric platform applies a constant potential at the working electrode related to the reference electrode, where the potential is obtained from the electrochemical oxidation or electroreduction of an electroactive species [48,49]. This system has several advantages, including low costs and sensitivity. It can be used in conjunction with mediators such as iodine or ferrocenedicarboxylic acid (FEDC), to improve their selectivity. Moreover, there is great potential for miniaturization of this system, which leads to smaller sample volume [50]. Over the years, amperometric immunosensors have been used to detect various pathogens. This system was used for the detection of *Escherichia coli* O157:H7 in food specimens. The method relies on first, the long-chain, amine-terminated alkanethiol 11-amino-1-undecanethiol hydrochloride (AUT) self-assembling on a gold electrode surface and providing an ordered, oriented, stable and compact monolayer for the immobilization of massive AuNPs. Next, chitosan-multiwalled carbon nanotubes–SiO2/thionine (CHIT–MWNTs–SiO2@THI) composite is synthesized and attached to an electrode surface. According to the results, *E. coli* O157:H7 was detected in milk and water samples with a limit of detection (LOD) at 2.5 × 10^2^ colony-forming unit (CFU)/mL [51]. The amperometric immunosensor system was used for the detection of *Mycobacterium tuberculosis* in sputum samples. In this study, an amperometric biosensor with microtip immunoassay was used. The system is based on a focused amperometric measurement produced by a high electric-field and concave-meniscus profile near the microtip area. Detection antibodies were specifically captured on the microtip area, and the electrical current was increased upon the capture of *M. tuberculosis*. The LOD was 1 × 10^2^ CFU/mL [52]. In a different study, a disposable enzyme-labeled amperometric immunosensor for *Listeria monocytogenes* detection was developed. The immunosensor was developed by immobilizing the horseradish peroxidase (HRP)-labeled antibody against *L. monocytogenes* onto the surface of the novel multiwalled-carbon-nanotube (MWCNT)-fiber electrode. This immunosensor exhibited acceptable reproducibility, specificity, and stability, and the detection limit was 1.07 × 10^2^ CFU/mL [53]. A label-free amperometric immunosensor for hepatitis-B-surface-Ag determination was also developed. The immunosensor was based on the immobilization of Ab molecules on a biocompatible redox-active poly(allylamine)-branched ferrocene (PAA-Fc)/AuNPs glassy-carbon electrode. The PAA-Fc composite retains its electrochemical activity, avoids the leakage of Fc, and enhances the conductivity of the composite. The AuNPs adsorption onto the PAA-Fc matrix provides sites for Ag immobilization and a favorable microenvironment for maintaining its activity. The method is efficient, cost-effective, potentially attractive for clinical immunoassays, and the LOD was 40 pg/mL [54]. The examples of the use of an amperometric immunosensor for pathogen detection are summarized in Table 1. 

#### 3.1.2. Potentiometric Platform 

Measuring the change of potential due to the formation of the immunocomplex between Ab and Ag is the principle of potentiometric immunosensors. In this method, the conversion of the biorecognition process into a change in potential signal is detected by a reference electrode [33]. The label-free potentiometric immunosensor for *Salmonella typhimurium* detection was developed. The immunosensor is based on the surface-blocking principle and a zero-current passive-ion-flux developed on a paper-based platform. A paper-strip ion-selective electrode with a carboxylated-polyviny- chloride (PVC)-membrane was integrated with a filter-paper pad, which acted as reservoir for the internal solution. The limit of detection was established on 5 cells/mL [58]. Silva et al. [59] applied a label-free potentiometric immunosensor toward *S. typhimurium*. The signal-output amplification was applied to a gold nanoparticle polymer-inclusion-membrane (AuNPs-PIM) that was used as a sensing platform and also for antibody immobilization. Moreover, a marker ion was used to detect the Ab-Ag binding event at the electrode surface. A detection limit of 6 cells/mL was attained. In another study, a silicon-chip-based light-addressable potentiometric sensor (LAPS) assay was utilized to detect *S. typhimurium*. Biotinylated and fluorescein-labelled anti-*Salmonella* Abs were selected as biorecognition elements. The sensitivity of this assay was approx. 1.19 × 10^2^ CFU/mL [60]. A different potentiometric immunoassay for the detection of enterovirus 71 (EV71) was also developed, using a silver (Ag+) ion-selective electrode (ISE). First, carboxylated dendrimer-doped AgCl nanospheres were synthesized and used to label mouse anti-EV71-detection pAbs using the carbodiimide coupling procedure. The immunoreaction was performed on an anti-EV71-capture mAb-coated microplate, using a biofunctional AgCl nanosphere as the detection Ab. This assay was carried out with a sandwich-type immunoassay format. The potential was monitored by using a digital ion-analyzer with a two-electrode system consisting of Na-ISE as the reference electrode and Ag-ISE as the working electrode. The LOD was established at 0.058 ng/mL [61]. A summary of the use of the potentiometric method in the detection of various pathogens is presented in Table 2.

#### 3.1.3. Impedimetric Platform

In this type of immunosensor, the impedance of the sensor is measured, which is affected by the biological reaction [63]. Electrochemical impedance spectroscopy (EIS) is an effective technique for the investigation of the formation of complexes among biomolecules on the surface of an electrode by probing the electrode/electrolyte interfacial properties. EIS measures a small sinusoidal-AC-voltage perturbation signal and measures the resulting AC current. These measurements are often fitted with the Randles equivalent-electric-circuit and impedimetric signal, which relies on the change in one of these equivalent-electric-circuit parameters upon analyte binding [64]. A microfluidic flow-cell with an embedded-gold interdigitated-array microelectrode (IDAM) was developed and integrated with magnetic nanoparticle-antibody conjugates (MNAC) into an impedance immunosensor for the purpose of *E. coli* O157:H7 detection. This system is able to detect 1.6 × 10^2^ in pure culture and 1.2 × 10^3^ cells of *E. coli* O157:H7 in ground-beef samples in 35 min [65]. In another study, a polydimethylsiloxane (PDMS) microfluidic-impedance immunosensor integrated with a specific Ab-immobilized alumina-nanoporous membrane was developed for detection of *Staphylococcus aureus*. The Abs were covalently immobilized onto nanoporous-alumina membranes via self-assembled (3-glycidoxypropyl) trimethoxysilane (GPMS) silane. The nanoporous alumina membrane is used in impedimetric immunosensing because of the increase in the electron-transfer through the electrode-solution interface caused by its high pore-density, biocompatibility and extension of surface area. This immunosensor provides e bacteria detection within 2 h with a high sensitivity of 1 × 10^2^ CFU/mL [66]. A nonstructural-Ab (NS1)-based impedimetric immunosensor, coupled with a bovine-serum-albumin (BSA)-modified screen-printed carbon electrode (SPCE) as the transducing substrate for the early diagnosis of dengue virus, were also developed. In this method, first, the anti-NS1 monoclonal Ab (mAb) is immobilized on the electro-grafted BSA surface of the working electrode. Then, the change in electron-transfer resistance with NS1 interaction is monitored, using EIS. This immunosensor successfully detected the dengue-virus protein with an LOD of 0.3 ng/mL [67]. An electrochemical-impedance immunosensor was also used to directly detect toxins, e.g., ricin. The nanoporous-aluminum substrate was hydrophobically modified via the self-assembled monolayer of 3-aminopropyltriethoxysilane (APTES). An immunosensor for the ricin detection was fabricated using the covalent cross-linking of Ab with self-assembled APTES. It detected the presence of ricin in milk, vegetable soup, and tomato juice containing 500 ng/mL of toxin in 20 min [68]. EIS can also be used for the detection of trace concentrations of Staphylococcal enterotoxin B (SEB). An anti-SEB Ab is attached to the nanoporous-aluminum surface using the APTES/glutaraldehyde coupling system. This immobilization technique allowed the fabrication of a highly stable and reproducible sensing device. Using this system, it is possible to determine the presence of SEB in concentrations as low as 10 pg/mL, in 15 min [69]. The application of the impedimetric method for different pathogens detection is shown in Table 3.

#### 3.1.4. Conductometric Platform 

The conductometric immunosensor is based on a relationship between the biorecognition and conductance events [70]. When the reaction between the biorecognition component and Ag occurs, the conductivity of the current flow or solution is changed, due to the change in the concentration of the ionic species [71]. The biological signal is converted to an electrical signal through a conductive polymer such as polyaniline, polyacetylene or polypyrrole [72]. Polyaniline is the most extensively used conductive polymer, due to its strong biomolecular interactions, good conductivity and environmental stability [73]. A conductometric immunoassay for hepatitis B surface Ag (HBsAg) was developed. The assay relied on the bio-electrocatalytic reaction on the microcomb-type electrode using double-codified nanogold particles as labels. The microcomb-type electrode was produced on a transducer covered with an ordered anti-HBs/protein A/nanogold-architecture. The double-codified nanogold particles were prepared by using nanogold-labeled anti-HBs Abs conjugated with horseradish peroxidase (HRP). The formation of the immunocomplex changed the direct electrical communication between the electrode and carried HRP, and thus local conductivity variations could be determined based on the bio-electrocatalytic reaction of the carried HRP. The described immunosensor exhibited a low detection limit of 0.01 ng/mL HBsAg [74]. An acetylcholinesterase-based conductometric biosensor was developed for the detection of aflatoxin B1 (AFB1). Acetylcholinesterase immobilized onto the surface of the conductometric transducer was used as the bio-selective element. The LOD of AFB1 was established at 0.05 μg/mL [75]. A conductimetric immunosensor incorporating a polyclonal Ab (pAb) sandwich-assay was developed, in which the Ab-detection labelled with polyaniline was developed for detecting *E. coli* O157:H7 and *Salmonella* spp. This immunosensor could detect 79 CFU/mL of *E. coli* O157:H7 and 83 CFU/mL of *Salmonella* spp. within 10 min [76]. A conductometric immunoassay based on magnetite nanoparticles for *E. coli* detection was also reported. The nanoparticles were directly immobilized on the conductometric electrode, using glutaraldehyde coupling. Biotinylated anti-*E. coli* Abs were immobilized on streptavidin-modified magnetite nanoparticles by biotin–streptavidin interaction. The incorporation of nanoparticles facilitated the increase in conductivity, allowing the detection of 0.5 CFU/mL of bacteria [77]. Table 4 summarizes the application of this method in pathogen identification.

### 3.2. Optical Immunosensors 

**Figure 3:**
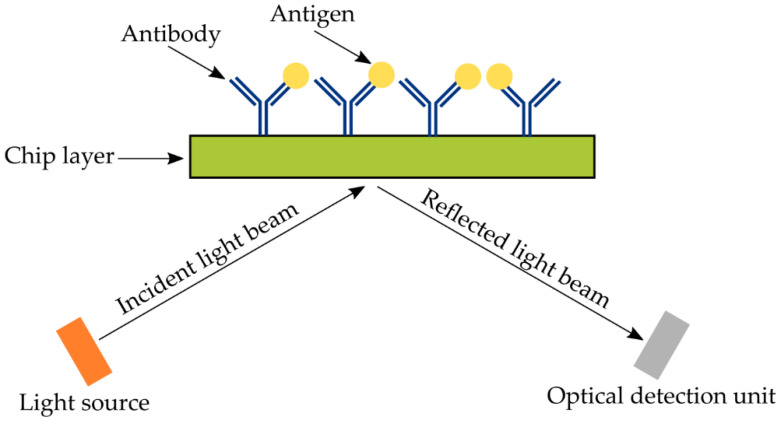
Schematic general principle of optical immunosensors.

The optical sensor system contains a light source, several optical components for generating a light beam with specific characteristics and directing this light to the modulating agent, a modified sensing head, and a photodetector [43]. Optical immunosensors can detect changes in optical properties in the evanescent field of an optical surface wave, in order to quantify Ag-Ab interactions. The evanescent field is generated when reflected and incident beams interfere with each other (Figure 3) [78]. 

Fiber-optic immunosensors are based on the measurement of fluorescent light excited by an evanescent wave generated by a laser to quantitatively detect biomolecules immobilized on the fiber surface [79]. This type of assay has been used to detect the *Clostridium botulinum* toxin. Abs specific for botulinum toxin were immobilized on the fiber surface. When the toxin bound to the surface, a second Ab labeled with tetramethylrhodamine-5-isothiocyanate (TRITC) was used for signal generation. Using the fiber evanescent wave, the binding events along the core of the tapered fiber were transduced as an increase in fluorescence intensity. The botulinum toxin was detected within a minute, at concentrations as low as 5 ng/mL [80]. The evanescent-wave fiber-optic biosensor for ricin detection was developed. A sandwich-immunoassay scheme was used to detect the ricin toxin. The avidin-coated fibers were incubated with biotinylated anti-ricin IgG to immobilize the Ab, using an avidin–biotin bridge. The LOD of ricin in river-water samples was established at 1 ng/mL. The entire assay was performed on previously prepared fibers within 20 min [81]. Another fiber-optic immunosensor to detect low levels of *Listeria monocytogenes* cells was developed. In this method, first, pAb is immobilized on polystyrene fiber waveguides through the biotin-streptavidin reaction, to capture bacteria cells on the fiber. Next, cyanine 5 (Cy5)-labeled murine mAb is used to generate a specific fluorescent signal. The sensitivity range is approx. 4.3 × 10^3^ CFU/mL for a pure culture of *L. monocytogenes* [82]. Morlay et al. [83] developed a label-free system based on surface plasmon resonance (SPR) imaging, which is an optical detection technique used to monitor and analyze biomolecular interactions in real time, coupled with an immunosensor specific to *L. monocytogenes* detection. A biochip covered with a gold layer was functionalized with different pAbs. During the analysis, the SPR signal was monitored in real time during the injection of different bacterial concentrations. The Ab successfully bound bacterial cells in lettuce samples inoculated with *L. monocytogenes* strains. This approach allowed the detection of a very small number of bacteria in foodstuff (from 17 to 25 CFU/25 g of lettuce). In a different study [84], an SPR sensor platform for *Campylobacter jejuni* detection was developed. SPR sensor chips were functionalized with pAbs against *C. jejuni* using covalent attachment, and then gold chips were applied for the direct detection of bacteria. Three different immunoassay-formats (direct, sandwich and sandwich-with-Ab-functionalized-AuNPs) were developed for the detection of *C. jejuni* on an SPR device. According to the results, the best immunoassay was the sandwich one, and the poorest was the direct immunoassay. The LOD obtained for the detection of bacterial cells using the sandwich immunoassay was 4 × 10^4^ CFU/mL. A sandwich SPR-immunosensor for the detection of SEB was also developed. The anti-SEB Abs were bound covalently onto the gold-chip surface, via attachment to carboxymethyl-dextran on the chip surface. The SPR-biosensor assay detected SEB at 10 ng/mL within 8 min [85]. The above-mentioned applications of the method are summarized in the Table 5.

### 3.3. Piezoelectric Immunosensors 

**Figure 4:**
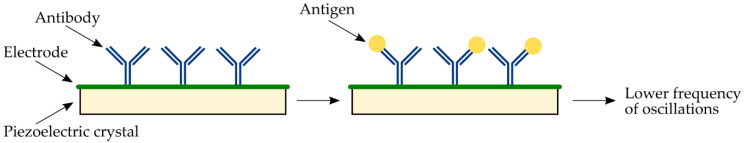
Schematic diagram of piezoelectric immunosensor.

Piezoelectric immunosensors are based on materials such as quartz crystals with Ab or Ag immobilized on their surface, and can be employed by the application of an external alternating-electric-field or by pH changing (Figure 4). The oscillation frequency is proportional to the change in quartz-crystal mass. The reaction between Ab and Ag (one immobilized on the surface and the other free in gas phase or solution), can be followed in real time. Factors such as effective viscosity, conductivity, electrode morphology, dielectric constant, density and temperature of the liquid, can also influence the frequency responses [43]. 

Oztuna et al. [86] presented an aminated-poly(vinyl chloride) (PVC-NH2) coated by the piezoelectric crystal immunosensor for the simultaneous, rapid detection of *Bacillus anthracis* spores. PVC-NH2 was used as an adhesive layer for mAb immobilization on gold quartz crystal [86]. Experiments conducted on primates showed that the estimated infective dose for *Bacillus anthracis* is 8000–50,000 spores [87]. The prepared immunosensor was tested in the range of infective doses mentioned above, and the detection limit was estimated as 2187 spores [86]. The immunosensing device based on a piezoelectric-sensor detection of the *Francisella tularensis* was also developed. The immunosensor included mouse pAbs immobilized in a layer of protein A covalently linked to the gold electrode of the sensor. The immunosensor is able to detect *F. tularensis* with a detection limit of 1×10^5^ CFU/mL in less than 5 min [88]. A piezoelectric immunosensor based on the amplification effect of the biotin-avidin system and the mass-multiplied effect of nano-gold particles was developed for abrin detection. The avidin is covalently attached to the biotin-labeled abrin pAbs, and is successfully immobilized to the gold electrode of the piezoelectric quartz crystal. The LOD was 0.05–5 mg/L [89]. A direct, label-free piezoelectric immunosensor was designed for the rapid detection of staphylococcal enterotoxin A (SEA), using quartz crystal microbalance with dissipation (QCM-D), as a transduction method. The sensing layer with the anti-SEA Ab was constructed using chemisorption of a self-assembled monolayer of cysteamine on the gold electrodes placed over the quartz crystal sensor, followed by the surface-amino-groups activation with the rigid homobifunctional cross-linker 1,4-phenylene diisothiocyanate (PDITC) and covalent linkage to the binding protein A. The LOD was 7 ng/mL for a total assay time of 25 min [90]. A direct and label-free immunoassay for SEB, based on a piezoelectric crystal immunosensor was fabricated. Three different immobilization methods were conducted: covalent immobilization based on the polyethyleneimine (PEI), covalent immobilization based on the self-assembled monolayer, and the protein A method. All of the immobilization methods used anti-SEB Abs undertaken on the gold-electrode PZ crystal on the gold electrode on one side of the piezoelectric crystal. The electrode coated with PEI showed the best results, and the self-assembled monolayer method provided the worst. The measurable range for SEB was 2.5–60 µg/mL, and the LOD was found to be 2.5 µg/mL [91]. Herein, we summarize the above-mentioned piezoelectric-immunosensor studies in the detection of different pathogens and toxins (Table 6).

## 4. Clinical Application of Biosensors and Real-Time Point-of-Care Test (POCT)

Biosensors have revolutionized diagnostics and medical care, and have also been used in many areas not directly related to medicine—environmental protection, food production, and even in bioterrorism countermeasures. The biosensor market is growing very dynamically. Analysts predict that its value may reach as much as USD 25 billion in 2021, and even USD 49.8 billion by 2030 [93]. Biosensors have been found in many applications in various fields, but they are probably the most widely used in medicine. In the previous decades, the highest percentage of biosensors was used in hospitals, in patient examination systems—from simple systems for monitoring blood glucose levels, to techniques for detecting tumor markers or the presence of viruses (e.g., HIV) in the body [94]. 

Point-of-care testing (POCT) as an innovative diagnostic technology began to be used on a large scale in medicine in the 1990s. POCT means not only diagnostics performed by the patient himself, but also quick diagnostics carried out by medical personnel in direct contact with the patient [95]. The use of POCT has become widespread in situations where large amounts of blood have to be drawn, to establish a diagnosis or to monitor treatment. This technique avoids iatrogenic anemia primarily in patients in intensive-care units, transplant patients, and especially in neonates and small children. The advantage of using POCT is the simplicity of the method. Medical personnel, such as nurses or paramedics, perceive the additional POCT obligation as not time-consuming [96]. Therefore, POCT in the form of biosensors is currently becoming one of the most important elements in the diagnosis of infectious diseases. An unquestionable advantage of using biosensors in the diagnosis of infectious diseases, apart from practicality, low invasiveness and low cost, is the possibility of their use a very short time after infection. In immunosensors, this time depends on the rate of production of specific antibodies, and therefore on the type of pathogen, whereas in nucleic-acid-based biosensors, the detection of a microorganism depends on the pathogen’s multiplication rate [97].

### 4.1. Bacterial Infection

Infectious diseases caused by pathogenic bacteria and those associated with high mortality require special attention from the public health system [98]. *Staphylococcus aureus*, *Escherichia coli* O157:H7, *Listeria monocytogenes*, *Salmonella typhimurium*, *Streptococcal bacteria*, *Mycobacterium tuberculosis*, *Bacillus cereus* and *Clostridium perfringens* are among the most common pathogens causing bacterial infections in humans [99]. The perennial increased antibiotics-administration, with both the incorrect use of antibiotics in the disease treatments that do not require antibiotic therapy, and their addition to food, cause the emergence of resistant bacteria [100]. Conventional diagnostic tools, although reliable, often require a lot of time and generate high costs, and very often effective treatment depends on quick diagnosis. Thus, the use of biosensors will on the one hand speed up the process, and on the other hand will be much cheaper [1].

*Staphylococcus aureus* is one of the most dangerous human pathogens. It demonstrates complex pathogenesis mechanisms, has a rich arsenal of virulence factors, and is also characterized by high resistance to chemotherapeutic-agent strains [101]. Methicillin-resistant strains of *S. aureus* (MRSA), described in 1961 [102], and resistant to all beta-lactam antibiotics except for the latest cephalosporins specifically targeting MRSA, proved to be particularly dangerous. Conventional microbial cultures typically require 3–5 days, while nucleic-acid technologies are very expensive, so the use of biosensors seems to be an option. Hernandez et al. proposed the use of a graphene-based potentiometric aptasensor to detect live *S. aureus* cells. This biosensor is made of a transducer layer (graphene oxide—GO or reduced GO—) and with a DNA aptameter attached to it (a sensing layer). These biosensors are characterized by high selectivity and sensitivity (detection of a single CFU/mL); however, sensors based on a reduced GO show a lower noise-level. The basis of the biosensor operation is the change in the recorded potential related to the preference of the aptameter binding to bacteria [103]. A potentiometric biosensor mainly for detecting *S. aureus* food contamination was developed by Ahari et al. Selective patterns for the *S. aureus* exotoxin are used in this biosensor, which enables the identification of bacteria. Although the main application of this sensor is in food-quality monitoring, it also has potential for medical diagnostics [104]. A slightly different approach was presented by Suaifan et al., who constructed a biosensor based on *S. aureus* proteolytic activity, to detect infections in healthcare settings. The biosensor is built of a specific peptide substrate located between the magnetic nanospheres and the gold-coated paper support. The basis of its operation is a color change visible to the naked eye, resulting from the dissociation of magnetic nanospheres-peptides in the presence of *S. aureus*, while the use of specialized software enables quantitative measurement (detection limit: pure-broth culture: 7 CFU/mL; inoculated in food products: 40 CFU/mL; inoculated environmental samples: 100 CFU/mL) [105]. 

*Escherichia coli* is a gram-negative, relatively anaerobic bacterium belonging to the *Enterobacteriaceae* family, which colonizes the human intestines and other warm-blooded animals [106]. *E. coli* is an opportunistic bacterium, but some *E. coli* pathogenic strains, together with *Salmonella enteritidis*, *Campylobacter jejuni, Shigella and Yersinia,* are responsible for the majority of bacterial diarrhea. One of the most dangerous enterohemorrhagic *E. coli* serotypes is O157:H7, which produces Shiga-like toxin, and may lead to the potentially fatal hemolytic-uremic syndrome (HUS) [107]. *Shigella*, like *E. coli*, also belongs to the *Enterobacteriaceae* family, and causes gastrointestinal infections. Thus, the development of fast, sensitive, and relatively cheap techniques aiming to detect and monitor this bacteria, is an important task for modern medicine. Wan et al. developed an impedimetric biosensor for the sensitive detection of *E. coli* O157:H7. This sensor is based on the transfer of electrons through a self-assembling monolayer through gold nanoparticles, onto which antibodies against *E. coli* have been transplanted. The attachment of gold nanoparticles to the surface of the bacteria resulted in a significant reduction of the electron-transfer resistance between the probe in the solution and the gold surface of the substrate [108]. In turn, Xiao et al. constructed fiber-optic biosensors, on which DNA probes capable of hybridizing to fluorescently labeled complementary-DNA were immobilized to identify *Shigella*. Importantly, the authors suggest that the sensitivity of this technique was comparable to the PCR method [109]. An optical genosensor for the early detection of *Shigella* was developed by Elahi et al. This technique utilizes the *Shigella* Spa gene, which was hybridized with the AuNP-DNA probe. The principle of the method is the change in color from red to purple in the absence of a complementary target, due to the aggregation of AuNP probes, in an acidic environment, while in the presence of a specific sequence (one of the four *Shigella* strains) it remains red [110]. 

Tuberculosis (TB) is one of the leading causes of death from infectious diseases worldwide. According to a WHO report from 2021, TB remains a major public-health threat worldwide, and the suboptimal global response to TB worsened during the COVID-19 pandemic [111]. The commonly used skin-test has low specificity, due to possible false-positive results in healthy subjects vaccinated with Bacillus Calmette–Guerin (BCG); thus, rapid and sensitive diagnostic techniques appear to play a key role in successful TB treatment. Rapid tests detecting both active TB and TB drug-resistance enable the patient’s diagnosis, regardless of the laboratory infrastructure or well-trained staff, leading to a reduction in delays in diagnostics, and thus a quick start to treatment. Importantly, the latest WHO guidance recommends the usage of Xpert MTB/RIF and Xpert MTB/RIF Ultra, in order to detect TB and rifamicin-resistance [112]. The Xpert MTB/RIF test is an automatic, fast (<2 h) nucleic-acid-amplification assay, consisting of a single-use multi-chambered pre-prepared cartridge. According to the WHO, the test material may be the sputum samples, but other biological samples are also possible: cerebrospinal fluid, urine, pleural fluid, ascetic fluid, dialysis fluid, and pus. To perform the test, staff may only be minimally trained, and biosafety cabinets are not required. Therefore, tests may be available in most basic diagnostic-laboratories [113]. The sensitivity and specificity of the test is presented in the updated Cochrane Review. Total sensitivity and complete specificity were shown to be 85% and 98%, respectively; sensitivity compared to microscopic smear is 61%, while the sensitivity and specificity for the detection of rifampicin-resistance are 95% and 99%, respectively [114]. In turn, the Xpert MTB/RIF Ultra (Xpert Ultra) test is an improved version of the Xpert MTB/RIF, which utilizes a newly developed cartridge and software [115]. A comparison of the sensitivity and specificity of both tests revealed that Xpert Ultra has a higher sensitivity (90.9% (86.2 to 94.7) vs. 84.7% (78.6 to 89.9)), but a lower specificity (95.6% (93 0.0 to 97.4) vs. 98.4% (97.0 to 99.3)) than Xpert MTB/RIF. Conversely, a comparison of the sensitivity and specificity for the detection of rifampicin-resistance revealed similar results for both tests [114].

### 4.2. Viral Infection

The global COVID-19 pandemic highlighted the key role of the rapid and universal availability of diagnostics tests for viral diseases, where biosensors are widely used. On the one hand, the use of biosensors accelerates the possibility of isolating potentially positive-patients and the possibility of giving appropriate treatment, and, on the other hand, it improves the comfort of the patient awaiting the result [97]. 

According to the WHO report of 4 May 2022, there have been a total of over 511 million cases of COVID-19 worldwide. Despite the fact that from the end of March 2022 there has been a decreasing trend in new cases of COVID-19, apart from in the African and the Americas regions, the problem remains very serious. The gold standard recommended by the WHO for the diagnostic of SARS-CoV-2 causing COVID 19, remains real-time PCR. Real-time PCR and other nucleic-acid tests are highly precise, but are time-consuming and require specialized laboratories and highly trained personnel. In turn, immunoassays widely used in care settings have a much lower sensitivity [116]. For this reason, the pursuit of new diagnostic techniques is very desirable. The use of nanomaterial-based biosensors may be a new approach. Park et al. developed a surface-plasmon-resonance biosensor for the rapid diagnosis of SARS, using the fusion reaction of gold-binding polypeptides with the virus surface-antigen (SCVme). The detection limit of 200 ng/mL was demonstrated, and the test time was 10 min [117]. In turn, Murillo et al. presented a test based on interferometric optical-detection for the identification of specific anti-SARS-CoV-2 immunoglobulins in saliva and serum, for the direct detection of antibodies, and requiring no signal enhancers or chemical triggers [118]. A dual-functional plasmonic biosensor combining the plasmonic photothermal effect and localized surface plasmon resonance (LSPR) has been proposed as an alternative technique for the detection of selected SARS-CoV-2 sequences. This sensor is characterized by high sensitivity, with a detection limit of 0.22 pM [119]. In contrast, Seo et al. developed a field-effect transistor (FET) sensor using graphene sheets coated with a specific antibody against the SARS-CoV-2 spike protein. The LOD was estimated at 1.6 pfu/mL and 2.42 × 10^2^ copies/mL for the culture medium and clinical samples, respectively [120]. Another biosensor proposal for SARS-CoV-2 screening is based on the detection of the SARS-CoV-2 S1 spike protein. This technique is characterized by a low detection limit: 1 fg/mL, a half-linear response range of 10 fg/mL–1 μg/mL, and a detection time of 3 min [121]. Similarly, the detection of the SARS-CoV-2 spike antigen was proposed by Krakus et al., and utilizes a colorimetric and electrochemical sensor based on gold nanoparticles. In the colorimetric method, as a result of contact with the antigen, gold nanoparticles changed their color from red to purple with a detection limit of 48 ng/mL. On the other hand, electrochemical detection was performed by spotting the probe solution on a disposable gold-electrode with screen printing, which enabled the detection of the SARS-CoV-2 antigen at the level of 1 pg/mL and a linear response to the antigen in the range of 1 pg/mL–10 ng/mL. Importantly, these techniques were specific to SARS-CoV-2, unlike the antigens of other pathogens including MERS COV, H1N1, and Streptococcus pneumoniae [122].

Despite reducing *Haemophilus influenza* infections as a result of the exacerbation of the COVID-19 pandemic, flu remains a serious problem. Hence, fast and critical diagnostics are still important. A label-free sensor that differentiates influenza A H1N1-subtypes (the seasonal and pandemic viruses: H1N1, H3N2 and 2009 H1N1) was developed by Bhardwaj et al. This biosensor relies on DNA aptamers by targeting the recombinant influenza-A-mini-hemagglutinin (mini-HA) protein. The sensitivity of this method (LOD) was found to be 3.7 plaque-forming units/mL [123]. In turn, Li et al. proposed a fluorescence sensor based on silver nanoparticles labeled with antibodies against H1N1. The LOD was estimated at 0.1 pg/mL, and the linear-detection range was 0.001–10 ng/mL [124]. Sensors detecting the serotype H5N1 were also designed [125,126]. The biosensor composed of a multi-functional DNA 3-way junction (3WJ) on a hollow Au spike-like nanoparticle (hAuSN) using an LSPR method, was presented by Lee et al. [125]. Meanwhile, Jiang et al. have developed a polydiacetylene-based biosensor for H5 influenza. The method of operation of this fast, sensitive (detection limit of 0.53 copies/μL) and specific biosensor, is to change the color from blue to red in the presence of the H5 virus. Moreover, using this technique it is possible to distinguish the H5 from the H3 influenza virus, the Newcastle disease virus and the porcine reproductive and respiratory syndrome virus [126].

The Ebola virus (EBOV) is an extremely virulent pathogen that causes epidemics of Ebola hemorrhagic fever, mainly in sub-Saharan African countries. In the years 2014–2016, EBOV spread to new areas, causing the largest epidemic of the disease in history and leading to the death of over 11,000 people. During this period, only 60% of cases were confirmed in the laboratory, which highlighted the need to seek for new fast and precise diagnostic tools [127]. An electrochemical DNA biosensor for the Ebola virus diagnosis has been proposed by Ilkhani and Farhad, the detection limit of which was 4.7 nM complementary oligonucleotides [128]. In turn, Baca et al. developed a surface-acoustic-wave biosensor for the Ebola virus that showed a limit of detection below the average level of viremia observed in the PCR test during the first day of symptomatic infection. A log-linear response was noted for highly fragmented Ebola viral-particles (detection limit—1.9 × 10^4^ PFU/mL, prior to virus inactivation). Moreover, it was suggested that the sensor would be more sensitive to the infectious Ebola virus in its intact form [129].

Human immunodeficiency virus (HIV) infection is a chronic disease that progressively reduces the immunity of an infected person. Early and prompt diagnosis is key to reducing both mortality and spreading in the population. Optical- or electrochemical-biosensors currently available for clinical usage are based on reactions which recognize and bind molecules on the surface, including antigen–antibody reactions, nucleic acid hybridization, enzyme-cofactor, etc., as well as viral-titer monitoring in nanoscale strategies [130]. Gong et al. demonstrated a DNA biosensor for the detection of a fragment of the HIV-1 gene, based on a polyaniline/graphene nanocomposite with a lower detection limit of 0.1 fM and log-linear response of 0.1 fM–0.1 nM [131]. The label-free biosensor for detecting HIV-1 was also designed by Lee et al. In this strategy for direct determination, a probe modified with gold nanoparticles was used, on which an antibody fragment was immobilized, and different concentrations of HIV-1 virus-like particles with a detection limit in the range of 600 fg/mL–375 pg/mL [132] were used. Moreover, Shafiee et al. presented a free-label optic biosensor, based on the early capture and quantification of HIV-1 by nanostructured photonic crystals with a detection range of 10^4^–10^8^ copies/mL [133].

The Zika virus, belonging to the Flaviviridae family, genus Flavivirus, is mainly found in Africa and South America, and is listed by the WHO as a pathogen with high pandemic potential. It was initially considered to be harmless. However, the increased number of infants born with encephalopathy in mothers infected by the Zika virus has led to increased attention to it. Fearing an epidemic, the search for quick, cheap and readily available diagnostic tests was started [134]. An electrochemical biosensor composed of surface-printed polymers and graphene oxide compounds has been proposed by Tancharoen et al. This strategy implies a correlation between the electrical-signal titers and the virus concentration in the solution (buffer/serum). Importantly, the detection limit with this biosensor is characterized by similar values to the RT-PCR [135]. In turn, Kaushik et al. presented an electrochemical immunosensor detecting the Zika virus based on a functionalized interdigitated gold-microelectrode on which antibodies specific for the virus envelope protein (Zev-Abs) were immobilized. This technology showed selectivity in relation to ZEV-ABS, and high sensitivity (12 kΩM -1). The detection range was 10 pM-1 nM, with a detection limit of 10 pM [136]. In contrast, Faria et al. developed a portable, easy-to-use and low-cost immunosensor based on ZnO nanostructures immobilized with the ZIKV-NS1 antibody on a printed circuit board using cystamine and glutaraldehyde. This strategy showed high selectivity, with a linear-detection range of 0.1–100 ng/mL and a detection threshold below 1 pg/mL. The undoubted advantage of this strategy is the use of undiluted urine as the test material, and the lack of cross-reaction with the Dengue virus surface-antigen [137]. 

Infectious diseases, especially those endemic to poorer parts of the world, could be potential sources of future pandemics. In order to accelerate the correct diagnostics and, consequently, the treatment, it is extremely important to develop low-cost, portable diagnostic-technologies. However, the strategies we propose above represent only a small percentage of the needs, because there is still no research on biosensors that can be used for many dangerous diseases, which implies the need to continue research in this area.

## 5. Conclusions 

Early detection of pathogens and toxins is essential and key for the rapid diagnosis and prevention of diseases. Various methods are widely used for their detection. Conventional laboratory-based methods such as plate culturing, ELISA and PCR techniques, remain dominant, but they have some disadvantages. As an alternative to conventional methods, the new approaches such as immunosensors have been developed and successfully applied in pathogen- and toxin-detection. Electrochemical, optical and piezoelectric immunosensors can detect pathogens such as *Escherichia coli*, *Salmonella typhimurium*, and *Mycobacterium tuberculosis* or toxins such as staphylococcal enterotoxin A, staphylococcal enterotoxin B, ricin, abrin, and botulinum neurotoxin, within minutes. Immunosensors possess great potential in becoming effective measurement tools, due to their real-time quantification, small sample-consumption, relatively low cost, and convenient instrument operation. It is believed that immunosensors will play a crucial role in the future pathogen and toxin sensor-detection. 

## Figures and Tables

**Table 1:** Application of amperometric immunosensor for pathogen detection.

Type of Immunosensor	Detected Pathogen	LOD	Reference
Electrochemical (Amperometric)	*Escherichia coli* O157:H7	2.5 × 10^2^ CFU/mL	[51]
	*Mycobacterium tuberculosis*	1 × 10^2^ CFU/mL	[52]
	*Listeria monocytogenes*	1.07 × 10^2^ CFU/mL	[53]
	Hepatitis B virus	40 pg/mL	[54]
	*Staphylococcus aureus*	1 CFU/mL	[55]
	*Salmonella typhimurium*	10 CFU/mL	[56]
	Ricin	10 ng/mL	[57]

**Table 2:** Application of potentiometric immunosensor for pathogen detection.

Type of Immunosensor	Detected Pathogen	LOD	Reference
Electrochemical (Potentiometric)	*Salmonella typhimurium*	5 cells/mL	[58]
	*S. typhimurium*	6 cells/mL	[59]
	*S. typhimurium*	1.19 × 10^2^ CFU/mL	[60]
	Enterovirus 71	0.058 ng/mL	[61]
	*Escherichia coli* O157:H7	7.1 × 10^2^ cells/mL	[62]

**Table 3:** Application of impedimetric immunosensor for pathogens detection.

Type of Immunosensor	Detected Pathogen	LOD	Reference
Electrochemical (Impedimetric)	*E. coli* O157:H7	1.6 × 10^2^ in pure culture 1.2 × 10^3^ cells	[65]
	*Staphylococcus aureus*	1 × 10^2^ CFU/mL	[66]
	Dengue virus	0.3 ng/mL	[67]
	Ricin	500 ng/mL	[68]
	Staphylococcal enterotoxin B	10 pg/mL	[69]

**Table 4:** Conductometric immunosensor in pathogen identification.

Type of Immunosensor	Detected Pathogen	LOD	Reference
Electrochemical (Conductometric)	Hepatitis B virus	0.01 ng/mL	[74]
	Aflatoxin B1	0.05 μg/ml	[75]
	*E. coli* O157:H7	79 CFU/mL	[76]
	*Salmonella* spp.	83 CFU/mL of	
	*E. coli*	0.5 CFU/mL	[77]

**Table 5:** Applications of optical immunosensors in the detection of different toxins and bacteria.

Type of Immunosensor	Detected Pathogen	LOD	Reference
Optical	*Clostridium botulinum* toxin	5 ng/mL	[80]
	Ricin	1 ng/mL	[81]
	*L. monocytogenes*	4.3 × 10^3^ CFU/mL	[82]
	*L. monocytogenes*	n/a	[83]
	*Campylobacter jejuni*	4 × 10^4^ CFU/mL	[84]
	Staphylococcal enterotoxin B	10 ng/ml	[85]

**Table 6:** Summary of research concerning the use of immunosensors in the detection of various pathogens and toxins.

Type of Immunosensor	Detected Pathogen	LOD	Reference
Piezoelectric	*Bacillus anthracis* spores	2187 spores	[86]
	*Francisella tularensis*	1 × 10^5^ CFU/mL	[88]
	Abrin	0.05 mg/L	[89]
	Staphylococcal enterotoxin A	7 ng/mL	[90]
	Staphylococcal enterotoxin B	2.5 µg/mL	[91]
	*Escherichia coli* O157:H7	10^3^ CFU/mL	[92]

## Data Availability

Not applicable.
